# Comprehensive characterization of glutamine synthetase-mediated selection for the establishment of recombinant CHO cells producing monoclonal antibodies

**DOI:** 10.1038/s41598-018-23720-9

**Published:** 2018-03-29

**Authors:** Soo Min Noh, Seunghyeon Shin, Gyun Min Lee

**Affiliations:** 1 Department of Biological Sciences, KAIST, 291 Daehak-ro, Yuseong-gu Daejeon, 34141 Republic of Korea; 2 The Novo Nordisk Foundation Center for Biosustainability, Technical University of Denmark, Hørsholm, Denmark

## Abstract

To characterize a glutamine synthetase (GS)-based selection system, monoclonal antibody (mAb) producing recombinant CHO cell clones were generated by a single round of selection at various methionine sulfoximine (MSX) concentrations (0, 25, and 50 μM) using two different host cell lines (CHO-K1 and GS-knockout CHO). Regardless of the host cell lines used, the clones selected at 50 μM MSX had the lowest average specific growth rate and the highest average specific production rates of toxic metabolic wastes, lactate and ammonia. Unlike CHO-K1, high producing clones could be generated in the absence of MSX using GS-knockout CHO with an improved selection stringency. Regardless of the host cell lines used, the clones selected at various MSX concentrations showed no significant difference in the GS, heavy chain, and light chain gene copies (*P* > 0.05). Furthermore, there was no correlation between the specific mAb productivity and these three gene copies (*R*^2^ ≤ 0.012). Taken together, GS-mediated gene amplification does not occur in a single round of selection at a MSX concentration up to 50 μM. The use of the GS-knockout CHO host cell line facilitates the rapid generation of high producing clones with reduced production of lactate and ammonia in the absence of MSX.

## Introduction

For large-scale production of therapeutic proteins including monoclonal antibodies (mAbs), recombinant Chinese hamster ovary (rCHO) cells are established using dihydrofolate reductase (DHFR)-based methotrexate (MTX) selection or glutamine synthetase (GS)-based methionine sulfoximine (MSX) selection^1^. The DHFR-based system, which has been extensively characterized with respect to gene amplification and production stability, has been the most widely used system because it results in high levels of gene amplification and expression^2–8^. However, it requires a multi-round of MTX selection for stepwise gene amplification, resulting in a longer timeline for cell line generation^9^. Thus, the use of the GS-based system, which requires a single round of MSX selection, is increasing due to a shorter timeline for cell line generation^10,11^. With a GS-knockout CHO cell line^12^ and promoter engineering, the GS-based system can be effective in cell line generation even in the absence of MSX^13^. Furthermore, the GS-based system mitigates ammonia accumulation in the culture medium because the overexpressed GS catalyzes the conversion of glutamate and ammonia into glutamine^14,15^.

Initially, the GS-based system used two rounds of MSX selection (25–50 μM MSX in the first round and 100–1000 μM for the second round for further gene amplification)^9,16–18^. Lately, a single round of MSX selection with a lower MSX concentration of 0–50 μM without further gene amplification is increasingly used because fewer copies of transgenes per cell are enough to achieve highly productive cell lines^13,19^. However, despite the increasing use of the GS-based system, rCHO cell line generation using the GS-based system has not been fully characterized with respect to gene amplification and production stability.

Unlike the DHFR-based system using a multi-round of selection with a stepwise increase of the MTX concentrations, it is questionable if a single round of selection at various MSX concentrations results in gene amplification. Furthermore, studies on the production stability of rCHO cell lines generated using GS-based MSX selection, which is one of the most important issues for the practical use of a cell line in industry, have not been fully substantiated yet^11,17,20,21^.

**Figure 1:**
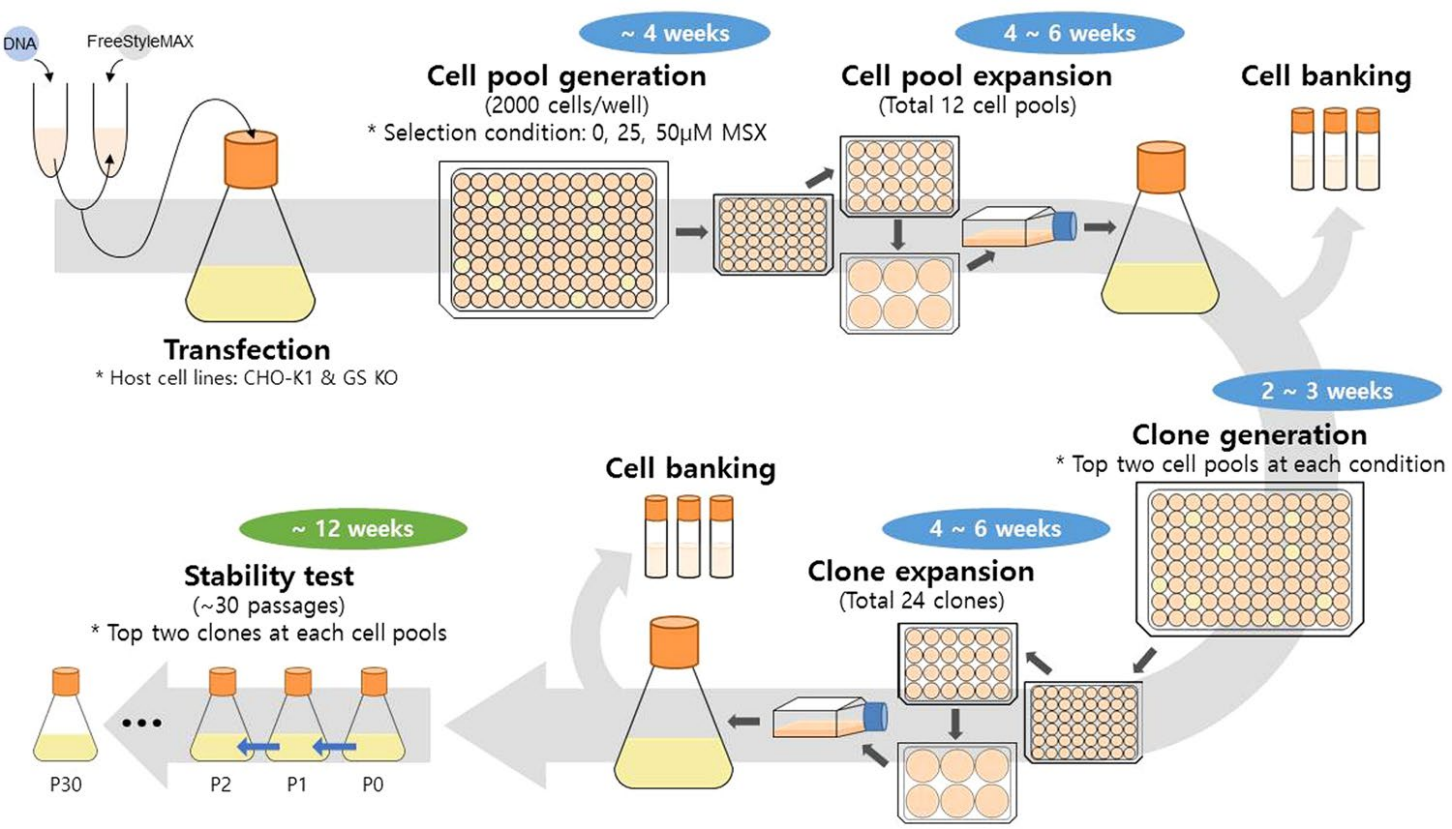
A schematic diagram of the process for mAb producing clone generation and long-term culture for testing the production stability.

In this study, we characterized the whole process of rCHO cell line generation using the GS-based MSX selection system. Using two different host cell lines (CHO-K1 and GS-knockout CHO (GS KO)), mAb producing rCHO cell clones were generated by a single round of selection at various MSX concentrations. A schematic diagram of the cell pool and clone generation process is shown in Fig. 1. Clones selected at each MSX concentration were analyzed with respect to the specific mAb productivity (*q*_mAb_), relative gene copy number, mRNA level, metabolites and amino acids utilization, and long-term production stability during 30 passages. Furthermore, clones generated by a single round of selection were subjected to a higher MSX concentration to evaluate the potential of GS-mediated gene amplification.

## Results

### Generation of mAb producing cell pools and clone

CHO-K1 and GS KO host cell lines were transfected with a vector containing *gs* and mAb genes as described earlier. The transfection efficiency was similar for both host cell lines (data not shown). After seeding the transfected cells at a concentration of 2000 cells/well into 96-well cell culture plates at various concentrations of MSX (0, 25, and 50 μM), the number of wells with one or more colonies was counted. The term “cell pool” indicates the cells survived in these 96-wells after selection, and “selection stringency” indicates the percentage of wells without a cell pool out of the total number of wells (600 wells) that were seeded with transfected cells.

**Figure 2:**
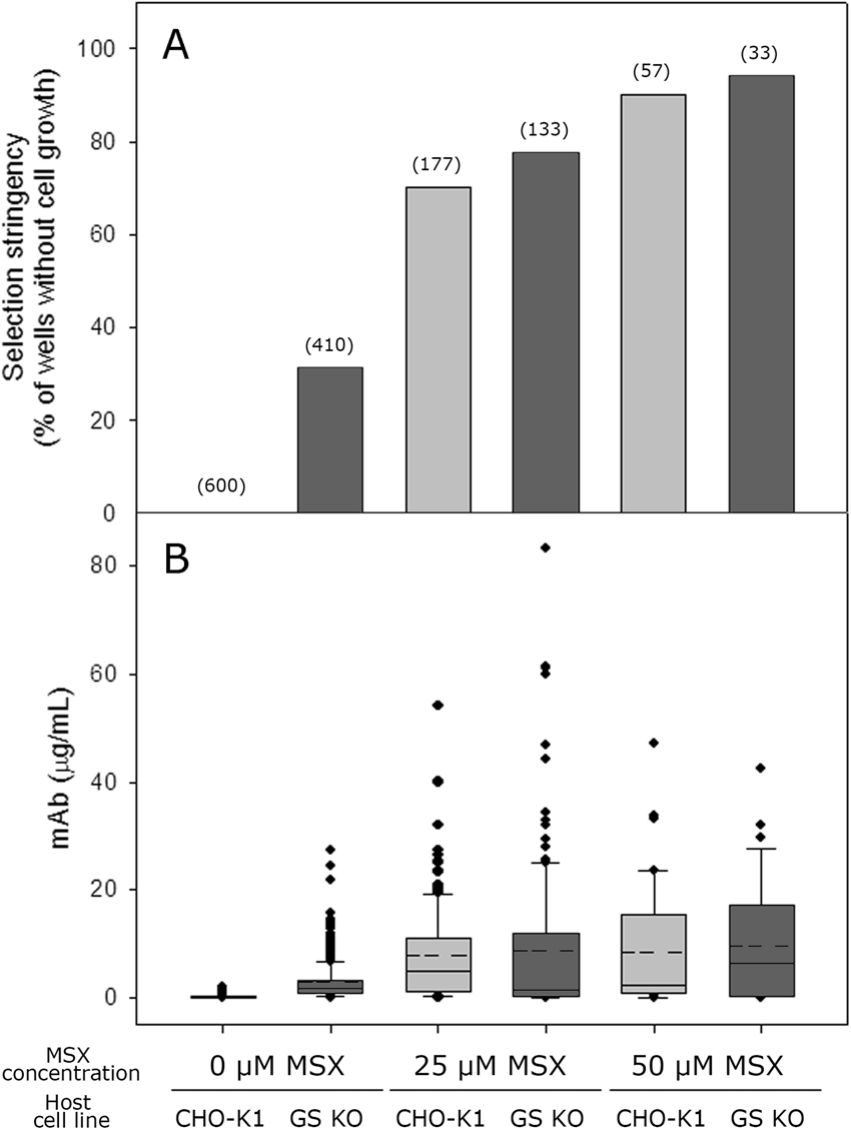
(**A**) Selection stringency and (**B**) mAb concentration of the culture supernatant in the wells with a cell pool at various MSX concentrations. Cell pools were derived either from CHO-K1 (light gray) or from GS KO (dark gray). The numbers in the parenthesis above the bar graphs indicate the number of wells with a cell pool. The box plot shows the mAb concentration of the culture supernatant in the wells with a cell pool. The dotted line in the box indicates the mean value, and the solid line in the box indicates the median value.

Figure 2 shows the selection stringency and mAb concentration of the culture supernatant in the wells with a cell pool at various MSX concentrations. For both the CHO-K1 and GS KO host cell lines, the number of wells with a cell pool decreased significantly with increasing MSX concentration but to different extents (Fig. 2A). For CHO-K1 cells, in the absence of MSX, all 600 wells contained a cell pool, whereas 177 wells contained a cell pool at 25 μM MSX. When the MSX concentration was further increased to 50 μM MSX, only 57 wells out of 600 wells contained a cell pool. The selection stringency was higher for GS KO than for CHO-K1 (Fig. 2A). For GS KO, only 410 wells out of 600 wells contained a cell pool in the absence of MSX. The number of wells with a cell pool decreased to 133 at 25 μM MSX and to 33 at 50 μM MSX. In addition, the GS KO-derived cell pools grew much slower than CHO-K1-derived cell pools. For the CHO-K1-derived cell pools in the absence of MSX, all wells showed cell pools within 20 days. In contrast, for the GS KO-derived cell pools in the absence of MSX, only 173 wells showed cell pools within 20 days.

As expected from the poor selection stringency of CHO-K1 in the absence of MSX, CHO-K1-derived cell pools showed little mAb production. The mAb concentrations in most wells in the absence of MSX were less than one μg/mL (Fig. 2B). The mAb production was significantly increased in the presence of MSX. The average mAb concentration was 7.8 μg/mL at 25 μM MSX. In contrast, the GS KO-derived cell pools showed a significant mAb production, more than 20 μg/mL in some wells, in the absence of MSX. The mAb production was further increased at 25 μM MSX, to more than 50 μg/mL in some wells. However, the differences in the average mAb concertation between CHO-K1 and GS KO at 25 μM MSX were not significantly different (*P* > 0.05). The average mAb concentrations of the GS KO-derived cell pools at 25 μM MSX were 9.8 μg/mL. Despite a more stringent selection at 50 μM MSX, the mAb production of the CHO-K1-derived and GS KO-derived cell pools was not increased further. The average mAb concentrations of the CHO-K1-derived and GS KO-derived cell pools at 50 μM MSX were 8.6 and 9.7 μg/mL, respectively.

The top two high producing cell pools at each condition were subjected to limiting dilution to establish single cell-derived clones. Because CHO cells are considered heterogeneous even after a very short period of cultivation^22,23^, the term “clone” would have been inappropriate. Nevertheless, throughout this article, the term “clone” indicates a single-cell derived cell population, which was generated by limiting dilution of a cell pool. The top two high producing clones from the top two high producing cell pools at various MSX concentrations, which were 24 clones in total, were selected for further analysis (Fig. 1).

### Analysis of mAb producing clones

As described earlier, a total of 24 clones were expanded for long-term cultures with the corresponding concentration of MSX. The first three batches in the long-term cultures were analyzed for their specific growth rate (μ) and *q*_mAb_. The relative gene copy numbers and relative mRNA levels of GS, heavy chain (HC) and light chain (LC) were analyzed with the first batch in the long-term cultures (Supplementary Table S1). The general trend of the analytical results was the same whether MSX was included in the culture media or not.

Regardless of the host cell lines used, clones selected at 50 μM MSX showed the lowest μ. The average μs of the CHO-K1-derived clones and GS KO-derived clones selected in the absence of MSX were 0.70 and 0.67/day, respectively, whereas those selected at 50 μM MSX were 0.58 and 0.56/day, respectively. Unlike μ, the CHO-K1-derived clones showed the highest average *q*_mAb_ at 50 μM MSX (23.55 pg/cell/day), whereas the GS KO-derived clones showed it at 25 μM MSX (28.16 pg/cell/day). Among the 24 clones, the top two highest producing clones were the GS KO-derived clones selected in the absence of MSX (KO-0 2–1 and KO-0 2–2), confirming that the use of GS KO as a host cell enables one to establish high producing cell lines in the absence of MSX.

For the CHO-K1-derived clones, clones selected at a higher MSX concentration showed a higher average value for the relative gene copy number for all three genes (GS, HC and LC), but the difference was not statistically significant (*P* > 0.05). Likewise, the GS KO-derived clones showed no significant correlation between the relative gene copy number and the MSX concentration used for selection (*P* > 0.05). In contrast, the relative mRNA level of all three genes in the CHO-K1-derived clones selected at 25 μM or 50 μM MSX was higher than that selected in the absence of MSX (*P* < 0.05). Unlike the CHO-K1-derived clones, the GS KO-derived clones showed no significant correlation between the relative mRNA level of GS and the MSX concentration used for selection (*P* > 0.05). Furthermore, the relative mRNA levels of HC and LC were the lowest in the GS KO-derived clones selected at 50 μM MSX.

**Figure 3:**
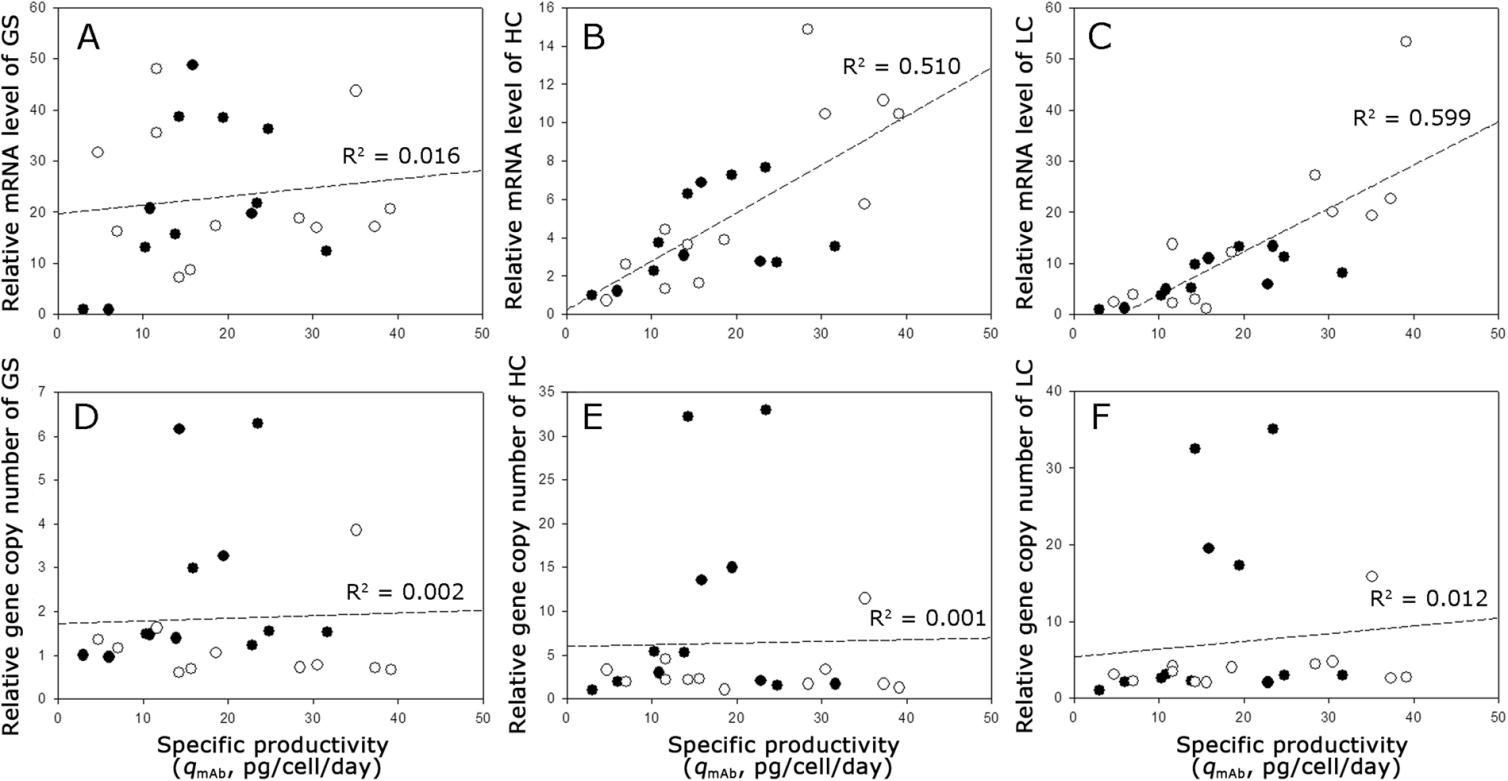
Correlation between (**A**) the relative mRNA level of GS and *q*_mAb_, (**B**) relative mRNA level of HC and *q*_mAb_, (**C**) relative mRNA level of LC and *q*_mAb_, (**D**) relative gene copy number of GS and *q*_mAb_, (**E**) relative gene copy number of HC and *q*_mAb_, and (**F**) relative gene copy number of LC and *q*_mAb_. The closed circles indicate the clones derived from CHO-K1, and the open circles indicate the clones derived from GS KO.

Figure 3 shows the correlation between the relative mRNA level and gene copy number of GS, HC, and LC and the *q*_mAb_ of all 24 clones selected at various MSX concentrations. While the relative mRNA level of GS was not related to the *q*_mAb_ (*R*^2^ = 0.016), the relative mRNA levels of HC and LC were meaningfully related to the *q*_mAb_ (*R*^2^ = 0.510 and 0.599, respectively) (Fig. 3A–C). However, there was no correlation between the *q*_mAb_ and the relative gene copy numbers for all three genes including HC and LC (*R*^2^ ≤ 0.012) (Fig. 3D–F). In fact, the top two highest producing clones (KO-0 2–1 and KO-0 2–2) had relatively low gene copy numbers for HC and LC. Thus, the clones with a high gene copy number for HC and LC did not necessarily show a high *q*_mAb_.

**Figure 4:**
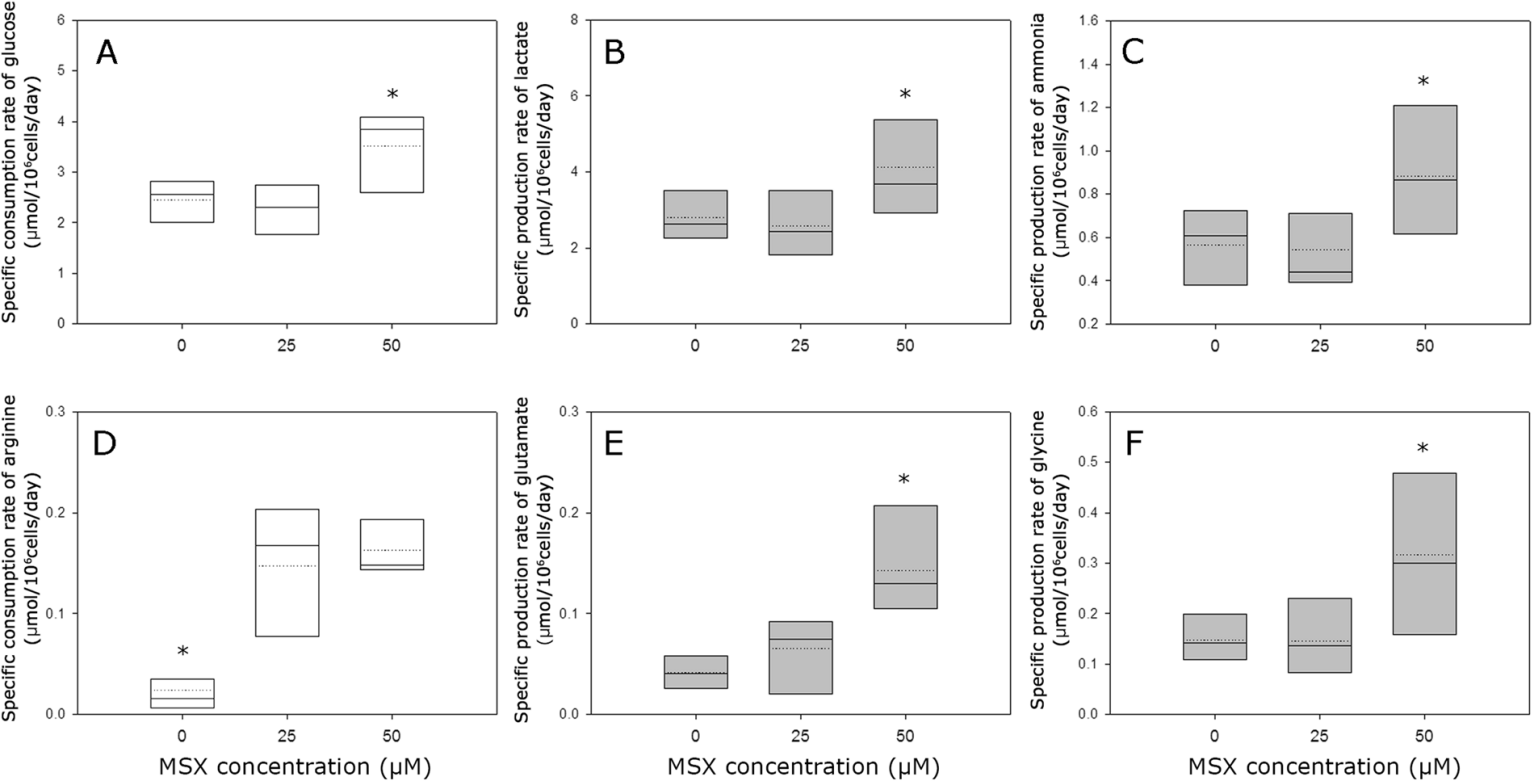
Specific consumption or production rate of (**A**) glucose, (**B**) lactate, (**C**) ammonia, (**D**) arginine, (**E**) glutamate, and (**F**) glycine. Clones from different selection conditions (0, 25 and 50 μM of MSX) are shown in the box plots. The dotted line indicates the mean value, and the solid line indicates the median value.

Figure 4 shows the consumption or production rates of metabolites and amino acids for all 24 clones selected at various MSX concentrations. Regardless of the host cell lines used, clones selected at the same MSX concentration showed no differences in the specific consumption or production rates except for the specific glycine production rate (*q*_gly_). The average *q*_gly_ of the CHO-K1-derived clones (0.14 μmol/10^6^cells/day) was significantly lower than that of the GS-KO-derived clones (0.27 μmol/10^6^cells/day) (*P* < 0.05). Clones selected at 50 μM MSX showed higher specific consumption or production rates of glucose, lactate and ammonia (*q*_glc_, *q*_lac_, and *q*_amm_, respectively) than those selected in the absence of MSX (Fig. 4A–C). In contrast, there was no significant difference in specific consumption rates of glutamine (*q*_gln_) at various MSX concentrations (0.02–0.14 umol/10^6^cells/day). Out of the 15 amino acids including glutamine, arginine, glutamate and glycine showed significantly increased specific consumption or production rates at 50 μM MSX compared with those in the absence of MSX (*P* < 0.05; Fig. 4D–F). Other amino acids, of which the specific consumption or production rates did not change at various MSX concentrations, are shown in Supplementary Fig. S1.

### Feasibility of GS-mediated gene amplification by stepwise selection for resistance to increasing concentrations of MSX

To determine whether a stepwise selection, not a single round selection, enables the amplification of gene copies in cells, 8 clones selected in the absence of MSX (four CHO-K1-derived clones (K1-01-1, K1-01-2, K1-02-1, and K1-02-3) and four GS KO-derived clones (KO-01-1, KO-01-3, KO-02-1, and KO-02-2)) were subjected to a higher concentration of MSX by subculturing them in culture medium containing 25 or 50 μM MSX. Cells were inoculated at a concentration of 1 × 10^6^ cells/mL into 125-mL Erlenmeyer flasks containing 30 mL of the culture medium. Every three days, cells were centrifuged at 1200 rpm for 5 min, and then, the spent culture medium was exchanged for fresh medium.

**Figure 5:**
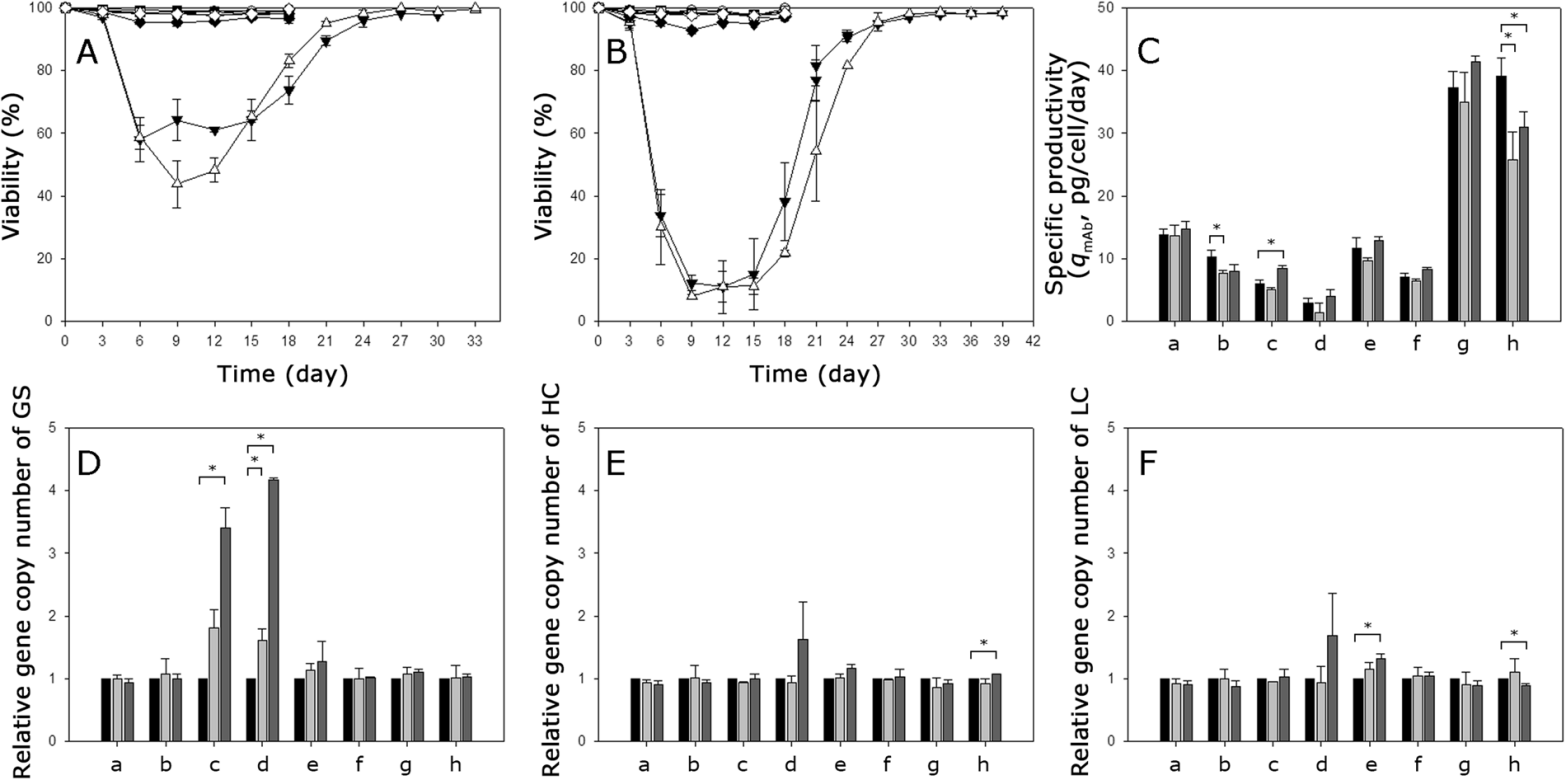
Feasibility test of GS-mediated gene amplification by stepwise selection for resistance to increasing concentrations of MSX. Viability change during the selection at (**A**) 25 μM MSX and at (**B**) 50 μM MSX. K1-0 1–1 (closed circles), K1-0 1–2 (open circles), K1-0 2-1 (closed triangles), K1-0 2–3 (open triangles), KO-0 1-1 (closed squares), KO-0 1–3 (open squares), KO-0 2–1 (closed diamonds) and KO-0 2–2 (open diamonds). Change in (**C**) *q*_mAb_, (**D**) relative gene copy number of GS, (**E**) relative gene copy number of HC, and (**F**) relative gene copy number of LC after further selection at 25 or 50 μM MSX. The gene copy number and mRNA level are relative to the values of the clones before further MSX selection. (a) K1-0 1–1, (b) K1-0 1–2, (c) K1-0 2-1, (d) K1-0 2–3, (e) KO-0 1–1, (f) KO-0 1–3, (g) KO-0 2–1 and (h) KO-0 2–2. Before selection (black), after selection at 25 μM MSX (gray) and after selection at 50 μM MSX (dark gray). Error bars represent the standard deviation determined by duplicate experiments. Asterisks (*) indicate a significant difference at *P* < 0.05.

Interestingly, 6 clones selected in the absence of MSX did not show any growth inhibition at a higher concentration of MSX. Only two out of the 8 clones (K1-0 2–1 and of K1-0 2–3) went through further selection with a higher level of MSX (Supplementary Fig. S2). A higher concentration of MSX imposed a stronger selection on these two clones. The viability of K1-0 2–1 went down to 58% at 25 μM MSX and to 11% at 50 μM MSX. Likewise, the viability of K1-0 2–3 went down to 44% at 25 μM MSX and to 8% at 50 μM MSX (Fig. 5A,B). However, the *q*_mAb_ of all 8 clones including K1-0 2–1 and of K1-0 2–3, regardless of their *q*_mAb_ values, did not change significantly after selection at a higher concentration of MSX (Fig. 5C).

Six clones, which showed no growth inhibition at 25 or 50 μM MSX, did not show any significant increase in gene copy numbers of GS, HC and LC (Fig. 5D–F). In contrast, the K1-0 2–1 and K1-0 2–3 clones, which underwent further selection with a higher concentration of MSX, showed a significantly increased GS gene copy number at 25 or 50 μM MSX. The extent of the increment in the relative GS gene copy number was positively correlated to the concentration of MSX. However, unlike the GS gene, the relative gene copy number of HC and LC in the K1-0 2–1 and K1-0 2–3 clones did not change at 25 or 50 μM MSX (*P* > 0.05). Thus, GS-mediated co-amplification of the LC and HC genes did not occur in all 8 clones subjected to 25 or 50 μM MSX.

### Long-term cultures of mAb producing clones in the presence or absence of selective pressure

To investigate the stability of the mAb producing clones during long-term cultures, a total of 24 clones were sub-cultured for 30 passages in the absence or presence of the corresponding concentration of MSX.

**Figure 6:**
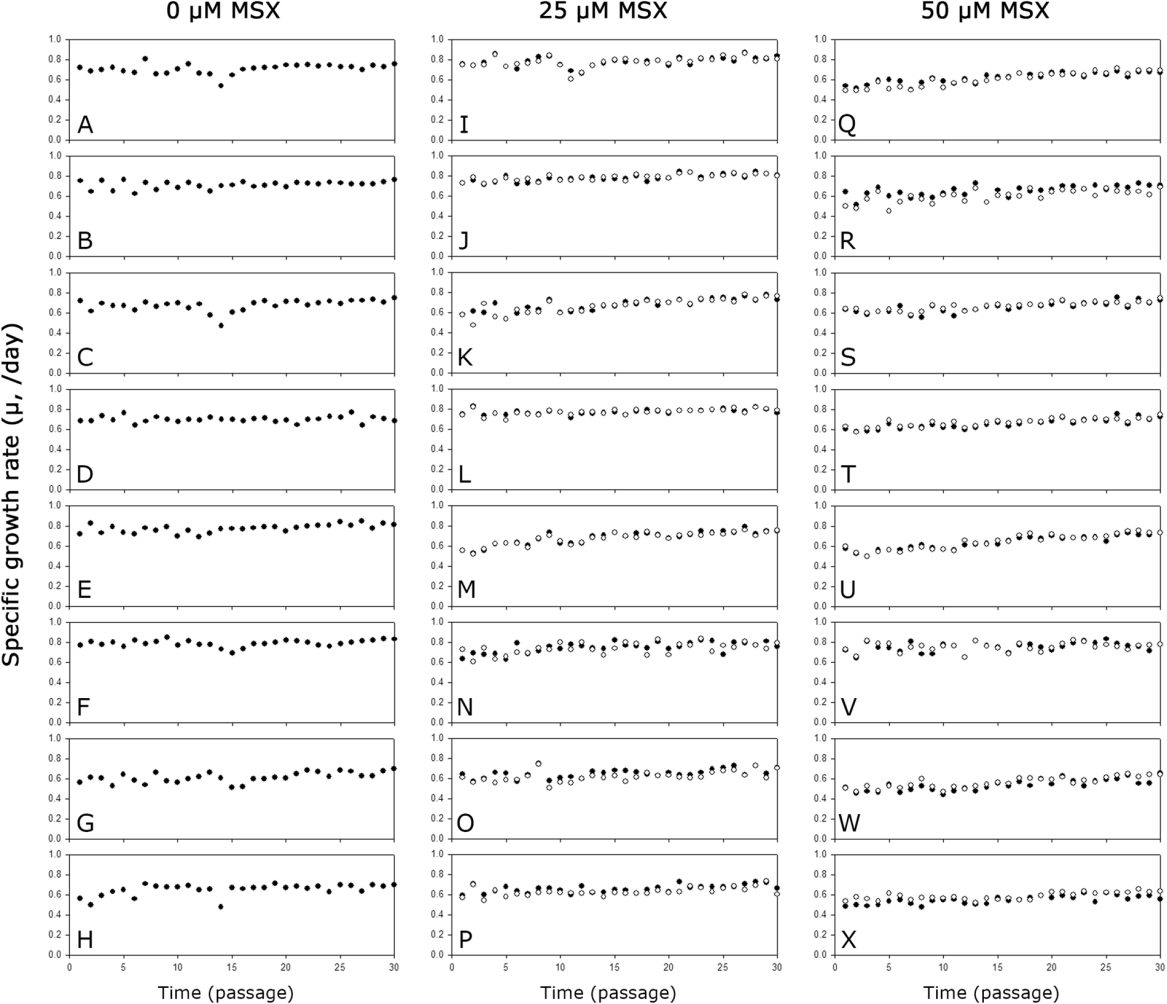
The μ of the mAb producing clones during long-term cultures with MSX (closed circle) or without MSX (empty circle). (**A**) K1-0 1–1, (**B**) K1-0 1–2, (**C**) K1-0 2-1, (**D**) K1-0 2–3, (**E**) KO-0 1–1, (**F**) KO-0 1–3, (**G**) KO-0 2-1, (**H**) KO-0 2–2, (**I**) K1-25 1–1, (**J**) K1-25 1–2, (**K**) K1-25 2-1, (**L**) K1-25 2–2, (**M**) KO-25 1–1, (**N**) KO-25 1–3, (**O**) KO-25 2-1, (**P**) KO-25 2–2, (**Q**) K1-50 1–2, (**R**) K1-50 1–3, (**S**) K1-50 2–1, (**T**) K1-50 2–2, (**U**) KO-50 1–1, (**V**) KO-50 1–3, (**W**) KO-50 2-1, and (**X**) KO-50 2–2.

Figure 6 shows the μ of the mAb producing clones during the long-term cultures. Regardless of the host cell lines used, the presence of MSX did not affect the μ significantly. Most clones showed an increase in μ during the long-term cultures but to different extents. Clones selected at a higher concentration of MSX showed a higher increment in μ during the long-term culture. When the first and last three passages were compared, the increase in μ at 0, 25, and 50 μM MSX were 8.6%, 15.2% and 22.0%, respectively (Supplementary Table S2).

**Figure 7:**
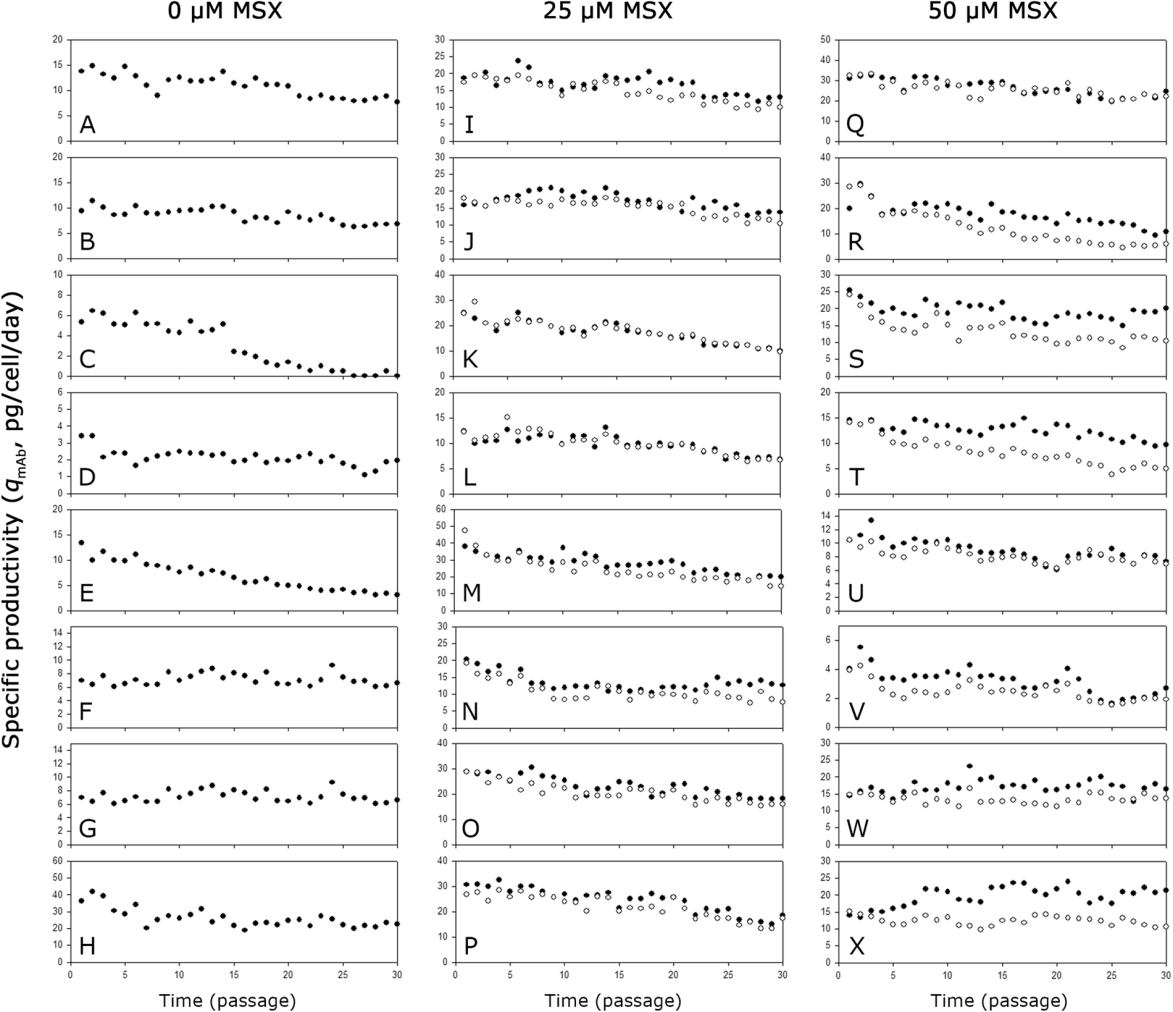
The *q*_mAb_ of the mAb producing clones during long-term cultures with MSX (closed circle) or without MSX (empty circle). (**A**) K1-0 1–1, (**B**) K1-0 1–2, (**C**) K1-0 2-1, (**D**) K1-0 2–3, (**E**) KO-0 1–1, (**F**) KO-0 1–3, (**G**) KO-0 2-1, (**H**) KO-0 2–2, (**I**) K1-25 1–1, (**J**) K1-25 1–2, (**K**) K1-25 2-1, (**L**) K1-25 2–2, (**M**) KO-25 1–1, (**N**) KO-25 1–3, (**O**) KO-25 2-1, (**P**) KO-25 2–2, (**Q**) K1-50 1–2, (**R**) K1-50 1–3, (**S**) K1-50 2–1, (**T**) K1-50 2–2, (**U**) KO-50 1–1, (**V**) KO-50 1–3, (**W**) KO-50 2–1, and (**X**) KO-50 2–2.

Figure 7 shows the *q*_mAb_ of the mAb producing clones during the long-term cultures. Regardless of the host cell lines and the MSX concentration used for selection, most clones showed a decreased *q*_mAb_ in a similar manner during the long-term cultures. In most clones, *q*_mAb_ decreased more significantly in the absence of MSX than in the presence of MSX (Supplementary Table S2). However, clones such as KO-0 1–3 and KO-50 2–1 showed a relatively stable *q*_mAb_ during the long-term cultures even in the absence of MSX.

To assess the degree of population heterogeneity during the long-term culture, the changes in the intracellular mAb content of the clones were measured by flow cytometry. CHO-K1 or GS KO was used as a negative control (Supplementary Fig. S3). In most clones, the changes in intracellular mAb contents, regardless of the presence of MSX, were not significant during long-term culture, suggesting that the decreased *q*_mAb_ was not mainly due to the occurrence of non-producers. Some clones such as K1-0 1–1, K1-50 1–3, and KO-50 1–3 showed a subpopulation with a lower intracellular mAb content during the long-term cultures.

To investigate whether the changes in the *q*_mAb_ of the clones were related to changes in their gene copy numbers of GS, HC and LC, the relative gene copy numbers were measured from gDNA samples prepared at passage 0, 10, 20, and 30 during the long-term cultures. Some clones showed a negligible change in the relative gene copy number while other clones showed a gradual decrease in the relative gene copy numbers during the long-term cultures (Supplementary Fig. S4).

**Figure 8:**
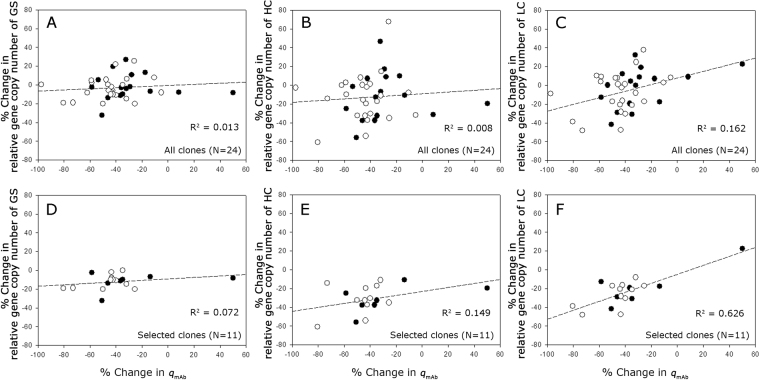
Correlation between (**A**) % change in the GS gene copies and % change in the *q*_mAb_ (all clones), (**B**) % change in the HC gene copies and % change in the *q*_mAb_ (all clones), (**C**) % change in the LC gene copies and % change in the *q*_mAb_ (all clones), (**D**) % change in the GS gene copies and % change in the *q*_mAb_ (selected clones), (**E**) % change in the HC gene copies and % change in the *q*_mAb_ (selected clones) and (**F**) % change in the LC gene copies and % change in the *q*_mAb_ (selected clones) during long-term cultures with MSX (closed circle) or without MSX (empty circle).

Figure 8 shows the correlation between the % change in the gene copy number of GS, HC, and LC and the % change in the *q*_mAb_ during the long-term cultures. When all 24 clones were used for the correlation analysis, there was no correlation between them (Fig. 8A–C). Among the 24 clones, 11 clones, which are denoted by the asterisk in Supplementary Figure S4, showed more than a 10% change in at least two genes among GS, HC, and LC. When these 11 clones were used for the correlation analysis, there was a meaningful correlation only between the % change in the LC gene copy number and the % change in *q*_mAb_ (Fig. 8D–F).

## Discussion

Due to the short timeline of the cell line generation, a GS-based MSX-selection system has been increasingly used for the establishment of rCHO cell lines. The MSX concentrations used for a single round of selection are usually 10–50 μM MSX for wild type CHO cell lines such as CHO-K1 and CHOK1SV^9,11,19,24,25^ and 0–25 μM for GS-knockout CHO cell lines with an improved selection stringency^12,13^. However, despite its popularity, the characterization of the GS-based selection system has not been fully substantiated yet. In particular, although GS-mediated gene amplification in rCHO cells was mentioned in numerous reports^11,16,17^, it is not clear that GS-mediated gene amplification really occurs in a single round of MSX selection. Furthermore, there have been conflicting reports on the production stability^11,17,20,21^. Therefore, we extensively characterized cell pools and clones generated using two different host cell lines, CHO-K1 and GS KO, according to the conventional procedure of cell line generation based on the GS-based selection system^12,19^.

As reported previously^12^, GS KO showed a higher selection stringency than that of CHO-K1. High producing cell pools and clones could be generated in the absence of MSX using GS KO. However, although the selection stringency increased further at 25 or 50 μM MSX, no dramatic increase in mAb production was achieved. In fact, the top two highest producing clones were the GS KO-derived clones selected in the absence of MSX. Furthermore, cell pools and clones were generated more rapidly in the absence of MSX compared with those in the presence of MSX. When compared with the CHO-K1-derived cell pools at 25 μM MSX, the GS KO-derived cell pools in the absence of MSX did not show an increased mAb production. Thus, the advantages of GS KO over CHO-K1 are a shorter timeline for cell line generation and no need of MSX for cell line generation and cell cultures.

When a total of 24 high producing clones generated at various MSX concentrations using the two different host cell lines were analyzed, there was no correlation between the *q*_mAb_ and the gene copy numbers of GS, HC and LC. These clones showed no significant correlation between the relative gene copy number and the MSX concentration used for selection, either (*P* > 0.05). Thus, regardless of the host cell lines used, selected clones did not undergo GS-mediated gene amplification in a single round of selection in the presence of MSX up to 50 μM. These results are consistent with previous reports^13,21^. The *q*_mAb_ was correlated meaningfully only with the mRNA levels of HC and LC. Thus, the transcriptional activity of the transgenes, which are subjected to rearrangement and are randomly integrated into host chromosomes, seems to be more important for the high level expression of both GS and target proteins rather than the number of integrated gene copies.

Unlike the GS-based system, the DHFR-based system, which typically has much higher gene copies up to 500, uses multiple rounds of MTX selection for a stepwise-increased gene amplification^6,7,26,27^. Thus, to see any potential of gene amplification through a stepwise increment of the MSX concentration, 8 high producing clones selected in the absence of MSX were exposed to 25 or 50 μM MSX. However, most clones did not undergo growth inhibition and gene amplification probably because their expression level of GS protein was high enough to overcome the inhibitory effect of MSX up to 50 μM. Only two clones underwent a further selection process at 25 or 50 μM MSX, and the relative gene copy number and mRNA level of GS increased proportionally with the increase of MSX concentration. However, the HC and LC genes were not co-amplified with the GS gene. The relative gene copy number of HC and LC as well as the *q*_mAb_ did not change even with the amplification of the GS gene. The genes of selection marker and gene(s) of a target protein were reported to be separated or partially deleted at high frequency in NS0 cells and CHO cells^28,29^. Co-amplification of genes may be achieved at a much higher level of MSX, but it is not worthwhile to pursue because of the severely depressed cell growth. Nevertheless, despite the stepwise increment of MSX, the gene amplification of HC and LC was not achieved at a MSX concentration up to 50 μM.

Cells that survived the GS-based selection adjusted their metabolism to survive with a balance between the amount of MSX in the media and the amount of overexpressed GS protein. Clones selected at 50 μM MSX showed different consumption or production rates for some metabolites and amino acids from those selected at 0 or 25 μM MSX. Such differences in their metabolism were the same regardless of the presence of MSX in the media. Thus, the metabolic changes in the clones selected at 50 μM MSX were not the temporary effect of MSX itself but were tuned during the selection process. Due to an increased *q*_lac_ and *q*_amm_, clones selected at 50 μM MSX produced increased levels of ammonia and lactate, which can have a negative effect on cell growth and product quality^30–34^. Therefore, the use of 50 μM MSX is again not recommended for the GS-based selection process.

Production stability is one of the most important criteria for the selection of rCHO cell lines in industry due to the inherent instability of CHO cells^22,35^. For the DHFR-based system, causes for the production instability during long-term cultures have been extensively studied, and the two major mechanisms are a loss of gene copy number^5,6^ and epigenetic gene silencing such as methylation^4,36^. For the GS-based system, information on production instability is somewhat limited. Possible causes for production instability in the GS-based system have been attributed to gene copy number and methylation^21^, lower cumulative cell time values and increased sensitivity to cellular stress^37^, and a gradual appearance of a secondary less producing population^20^. In this study, to examine the production stability of the 24 high producing clones selected at various MSX concentrations, cells were continuously sub-cultured for 30 passages, which was determined by considering the manufacturing processes of scale up from a frozen vial to a production-scale manufacturing process^38^. Regardless of the host cell lines and the MSX concentration up to 50 μM used for selection, the *q*_mAb_ of most clones decreased during 30 passages. Despite the decreased *q*_mAb_, 13 clones showed no change in the relative gene copy number, indicating a mechanism other than loss of gene copy number is responsible for the production instability in those clones. Such a weak correlation between the changes of the relative gene copy numbers and *q*_mAb_ also suggest that these high producing clones did not undergo GS/MSX-mediated gene amplification in a single round of selection.

In conclusion, GS-mediated gene amplification did not occur in high producing clones, which were generated in a single round of MSX selection up to 50 μM using CHO-K1 and GS KO host cell lines. In these clones, there was no correlation between the HC and LC gene copy numbers and the *q*_mAb_ or production stability during the long-term culture. Due to the improved selection stringency, high producing clones could be generated in the absence of MSX using the GS KO host cell line, which was accompanied with a rapid cell line generation and reduced production of toxic metabolites such as ammonia and lactate.

## Methods

### Host cell lines and culture maintenance

CHO-K1 (ATCC, Manassas, VA) and GS KO cell lines were used as host cell lines for mAb expression. The GS KO cell line was established in our laboratory by knocking out the GS gene (*gs*) in CHO-K1 using the transcription activator-like effect nuclease technology. Host cell lines were cultured in PowerCHO-2CD medium (Lonza, Basel, Switzerland) supplemented with 4 mM glutamine.

### Cell line development and culture maintenance

CHO-K1 and GS KO were transfected with a vector containing *gs* and mAb genes using FreeStyle^TM^ MAX (Thermo Fisher Scientific, Waltham, MA) according to the manufacturer’s protocol. After transfection, selection was carried out by seeding 2000 cells/well in 96-well cell culture plates containing selection media (a mixture of 20% PowerCHO-2CD and 80% ExCell CHO cloning medium (Sigma-Aldrich, St. Louis, MO) with GS expression medium supplement (GSEM, Sigma-Aldrich), 300 μg/mL zeocin, and various concentrations of MSX (0, 25, and 50 μM, Sigma-Aldrich)). mAb concentrations of the culture supernatants in the wells with one or more colonies, defined as a cell pool, were measured when the cell colonies covered more than 25% of the confluency. High producing cell pools were gradually expanded to grow in 125-mL Erlenmeyer flasks (Corning, Corning, NY). To construct single cell-derived clones, the top two high producing cell pools were subjected to limiting dilution using selection media supplemented with 5% dialyzed fetal bovine serum (Thermo Fisher Scientific). The top two high producing clones derived from each cell pool were used for long-term cultures.

### Cell culture

mAb producing cell pools and clones were cultured in PowerCHO-2CD medium supplemented with GSEM and various concentrations of MSX (0, 25 and 50 μM). Exponentially growing cells were inoculated at a concentration of 3 × 10^5^ cells/mL into 125-mL Erlenmeyer flask containing 50 mL of culture medium. The flasks were incubated in a Climo-shaking incubator (Kuhner Ag, Basel, Switzerland) at 110 rpm in a humidified 5% CO_2_/air mixture at 37 °C. Viable cells were distinguished from dead cells using the trypan blue dye exclusion method, and the viable cell concentration (VCC) was estimated using a Countess II^®^ FL automated cell counter (Thermo Fisher Scientific).

The long-term culture was done by continuous sub-culturing of the mAb producing clones for 30 passages (a period of approximately three months). Exponentially growing cells were inoculated at a concentration of 3 × 10^5^ cells/mL into 125-mL Erlenmeyer flask containing 30 mL culture medium and were sub-cultured every three days. At every passage, the VCC was evaluated, and culture supernatants were collected and kept frozen at −70 °C for further analyses. At passage 0, 10, 20 and 30, genomic DNA and total RNA were prepared for the analysis of the relative gene copy number and mRNA level.

### Measurement of mAb, metabolites, and amino acids

The secreted mAb in the culture supernatant was quantified using an enzyme linked immunosorbent assay as described previously^7^. The glucose, lactate, glutamine and ammonia concentrations were measured using a metabolite analyzer (Cedex Bio, Roche, Basel, Switzerland). Amino acids were analyzed by high-performance liquid chromatography using the Agilent 1200 series system equipped with the ACCQ-TAG 3.9 × 150 mm (Waters, Milford, MA) as described previously^39^.

The *q*_mAb_ and specific consumption or production rates of glucose, lactate, ammonia and each amino acid were evaluated on a plot of the substrate and product concentrations against the time integral values of the VCC^40^.

### Preparation of genomic DNA, total RNA, and cDNA

DNA was extracted from 5 × 10^6^ viable cells using Exgene^TM^ (GeneAll, Seoul, Korea) according to the manufacturer’s protocol. Total RNA was extracted from 5 × 10^6^ viable cells using Hybrid-R^TM^ (GeneAll) according to the manufacturer’s protocol. DNA and RNA concentrations were quantified using the NanoDrop^TM^ 2000 Spectrophotometer (Thermo Fisher Scientific). The extracted RNA was subsequently used as a template for cDNA using High Capacity cDNA Reverse Transcription Kits (Applied Biosystems, Foster City, CA) according to the manufacturer’s protocol.

### Analysis of relative gene copy numbers and mRNA levels

The genomic DNA samples and cDNA samples were prepared by diluting in distilled water, and qRT-PCR was performed on the CFX96 Real-Time System (Bio-Rad, Hercules, CA) using iQ™ SYBR® Green Supermix (Bio-Rad) according to the manufacturer’s protocol. Relative gene copy number and mRNA level of the GS, HC and LC were measured. PCR protocols and primers appropriate for each gene are shown in Supplementary Table S3. The data collected were analyzed using the 2^−ΔΔCt^ method^41^. The quantity of a target gene was calculated by normalization to GAPDH.

### Statistical analysis

The results are expressed as the mean ± standard deviation (SD) unless specified. When necessary, data were analyzed by Student’s t-test or one-way ANOVA test. The difference between the means was considered statistically significant at *P* < 0.05.

### Data availability

All data generated or analyzed during this study are included in this published article (and its Supplementary Information files). The datasets generated during and/or analyzed during the current study are available from the corresponding author on reasonable request.

## Electronic supplementary material

Supplementary Info
