# Supplementary material for: Comprehensive characterization of glutamine synthetase-mediated selection for the establishment of recombinant CHO cells producing monoclonal antibodies
Source: Sci Rep. 2018 Mar 29;8:5361. doi: 10.1038/s41598-018-23720-9 (PMC5876325; doi:10.1038/s41598-018-23720-9)
Supplement: Supplementary file 1 — Supplementary Info [file 41598_2018_23720_MOESM1_ESM.pdf]

Supplementary Information

Comprehensive characterization of glutamine synthetase-mediated selection for the establishment of recombinant CHO cells producing monoclonal antibodies

Soo Min Noh[1], Seunghyeon Shin[1] and Gyun Min Lee[1,2]

[1]Department of Biological Sciences, KAIST, 291 Daehak-ro, Yuseong-gu, Daejeon 34141, Republic of Korea; telephone: +82-42-350-2618; fax: +82-42-350-2610; e-mail: gyunminlee@kaist.ac.kr

[2]The Novo Nordisk Foundation Center for Biosustainability, Technical University of Denmark, Hørsholm, Denmark

*Correspondence to: Gyun Min Lee

Supplementary Figure S1. Specific consumption or production rate of (A) alanine, (B) asparagine, (C) glutamine, (D) isoleucine, (E) leucine, (F) lysine, (G) methionine, (H) phenylalanine, (I) proline, (J) threonine, (K) tyrosine, and (L) valine. Clones from different selection conditions (0, 25 and 50 μM of MSX) are shown in the box plots. The dotted line indicates the mean value, and the solid line indicates the median value.

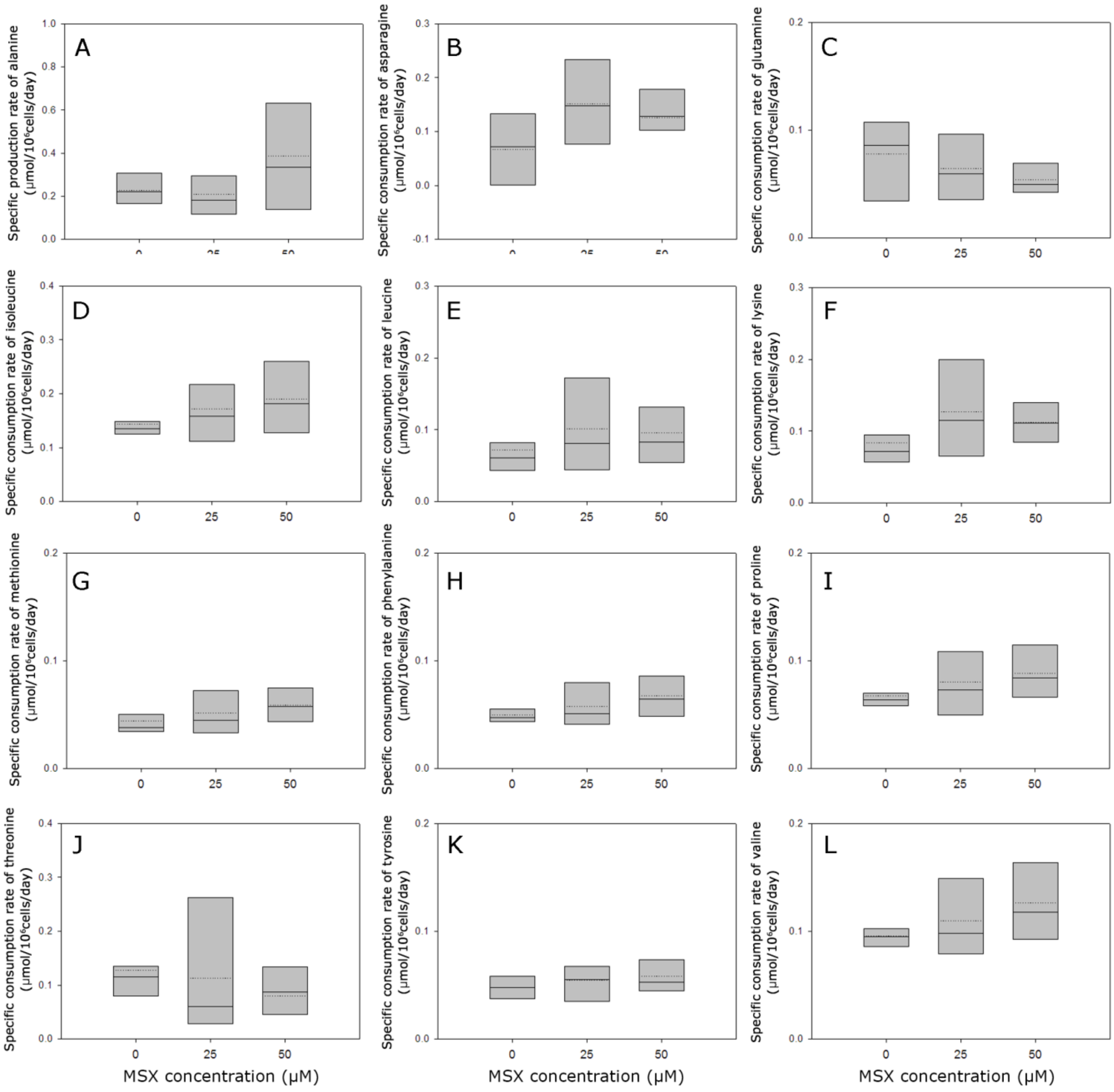

Supplementary Figure S2. Selection process of the clones at 25 μM MSX. (A) K1-0 1-1, (B) K1-0 1-2, (C) K1-0 2-1, (D) K1-0 2-3, (E) KO-0 1-1, (F) KO-0 1-3, (G) KO-0 2-1, and (H) KO-0 2-2, and at 50 μM MSX; (I) K1-0 1-1, (J) K1-0 1-2, (K) K1-0 2-1, (L) K1-0 2-3, (M) KO-0 1-1, (N) KO-0 1-3, (O) KO-0 2-1, and (P) KO-0 2-2. Viable cell concentration (open circle) and viability (closed circle). Error bars represent the standard deviation determined by duplicate experiments.

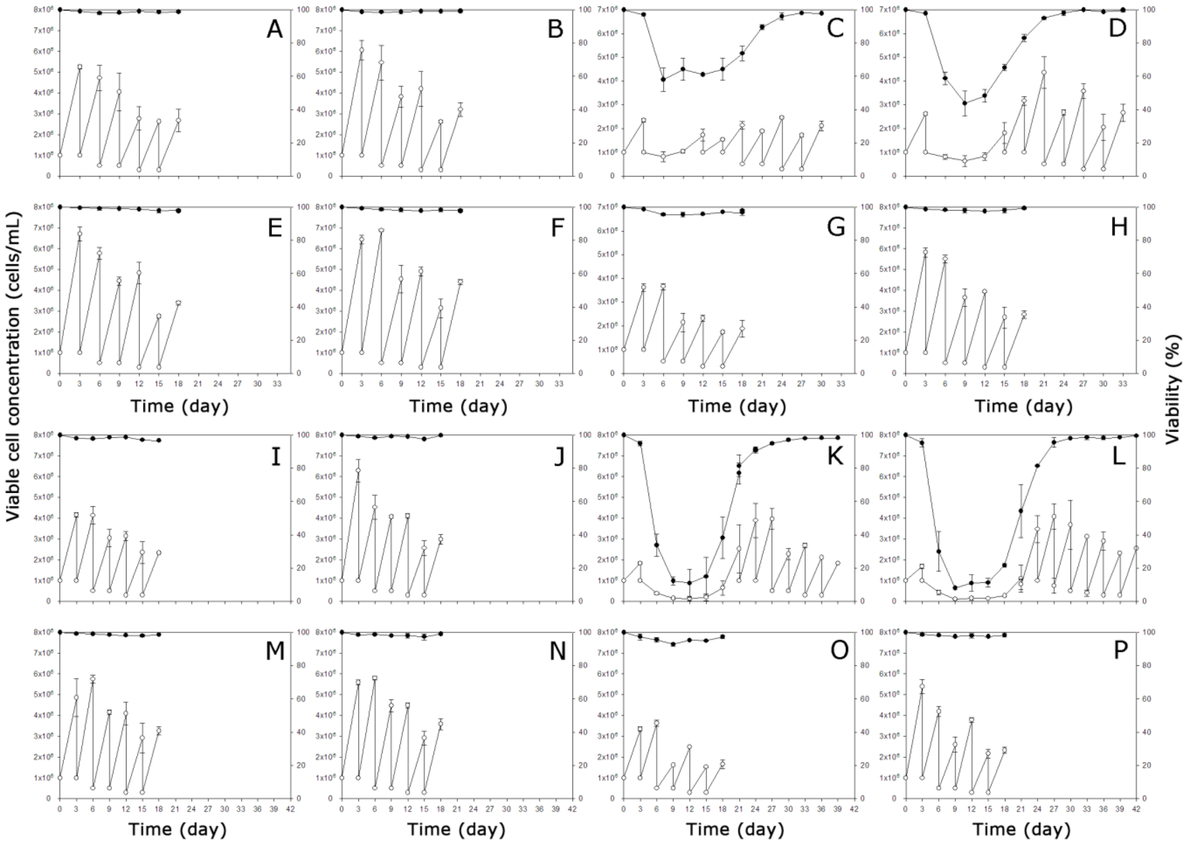

Supplementary Figure S3. Flow cytometric analysis of the intracellular IgG content during long-term cultures of (A) K1-0 1-1, (B) K1-0 1-2, (C) K1-0 2-1, (D) K1-0 2-3, (E) KO-0 1-1, (F) KO-0 1-3, (G) KO-0 2-1, (H) KO-0 2-2, (I) K1-25 1-1, (J) K1-25 1-2, (K) K1-25 2-1, (L) K1-25 2-2, (M) KO-25 1-1, (N) KO-25 1-3, (O) KO-25 2-1, (P) KO-25 2-2, (Q) K1-50 1-2, (R) K1-50 1-3, (S) K1-50 2-1, (T) K1-50 2-2, (U) KO-50 1-1, (V) KO-50 1-3, (W) KO-50 2-1, and (X) KO-50 2-2. The relative level of intracellular mAb was determined by flow cytometry using the cell analyzer LSR Fortessa™ (BD Biosciences, Franklin Lakes, NJ). To avoid day-to-day variation in the flow cytometric measurement, cell samples were fixed using 70% ethanol, kept at −20°C, and analyzed at the same time. For intracellular IgG content measurements, phycoerythrin-labeled goat anti-human IgGs specific for Fc gamma (eBioscience, Affymetrix, Santa Clara, CA) were used.

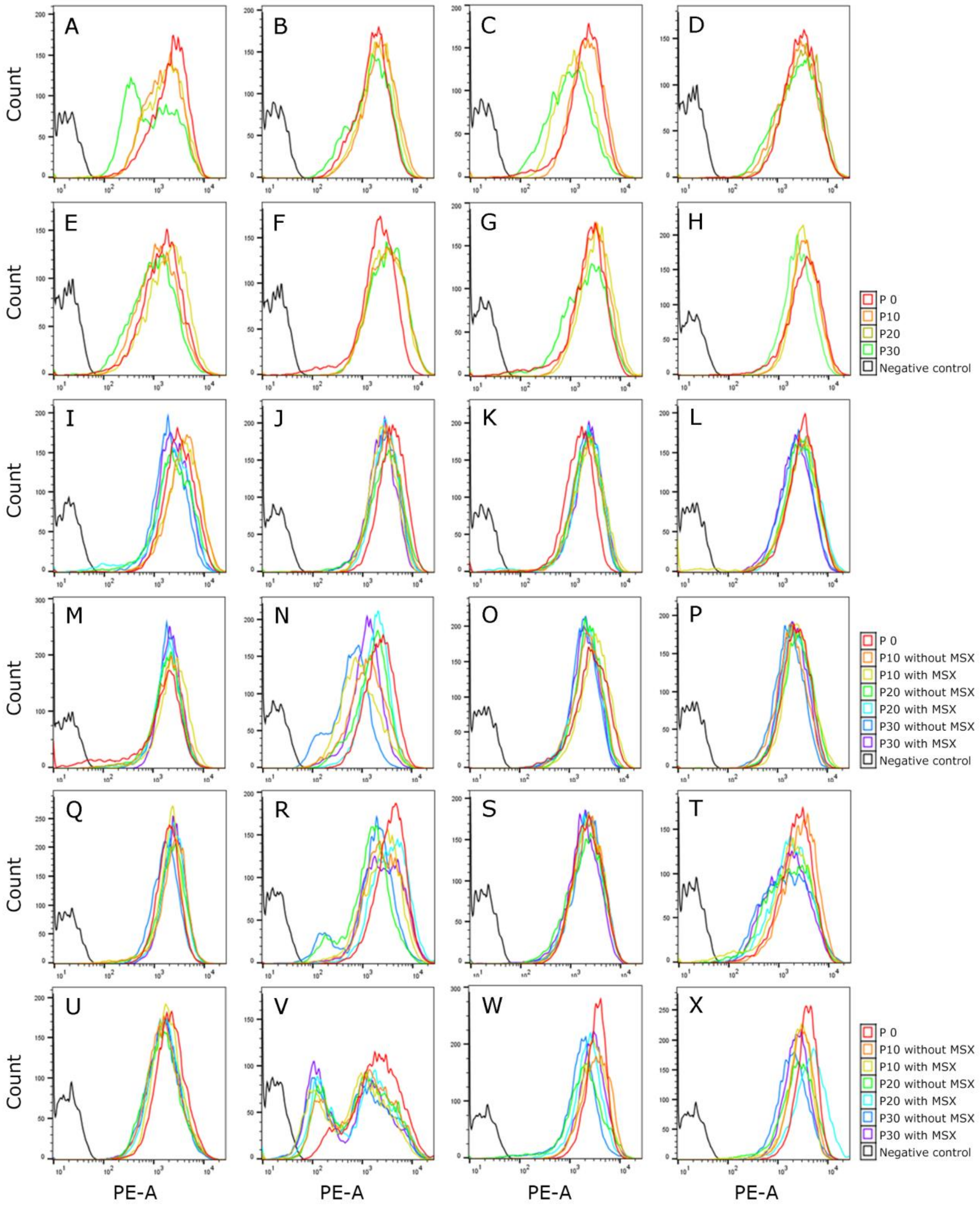

Supplementary Figure S4. Change in the relative gene copy numbers during the long-term culture of (A) K1-0 1-1, (B) K1-0 1-2, (C) K1-0 2-1, (D) K1-0 2-3, (E) KO-0 1-1, (F) KO-0 1-3, (G) KO-0 2-1, (H) KO-0 2-2, (I) K1-25 1-1, (J) K1-25 1-2, (K) K1-25 2-1, (L) K1-25 2-2, (M) KO-25 1-1, (N) KO-25 1-3, (O) KO-25 2-1, (P) KO-25 2-2, (Q) K1-50 1-2, (R) K1-50 1-3, (S) K1-50 2-1, (T) K1-50 2-2, (U) KO-50 1-1, (V) KO-50 1-3, (W) KO-50 2-1, and (X) KO-50 2-2. The gene copy numbers are relative to the values for the samples at passage 0 (P0). Asterisks (*) indicate clones which showed more than a 10% change in at least two genes among GS, HC, and LC during the long-term cultures. Error bars represent the technical error for the analysis.

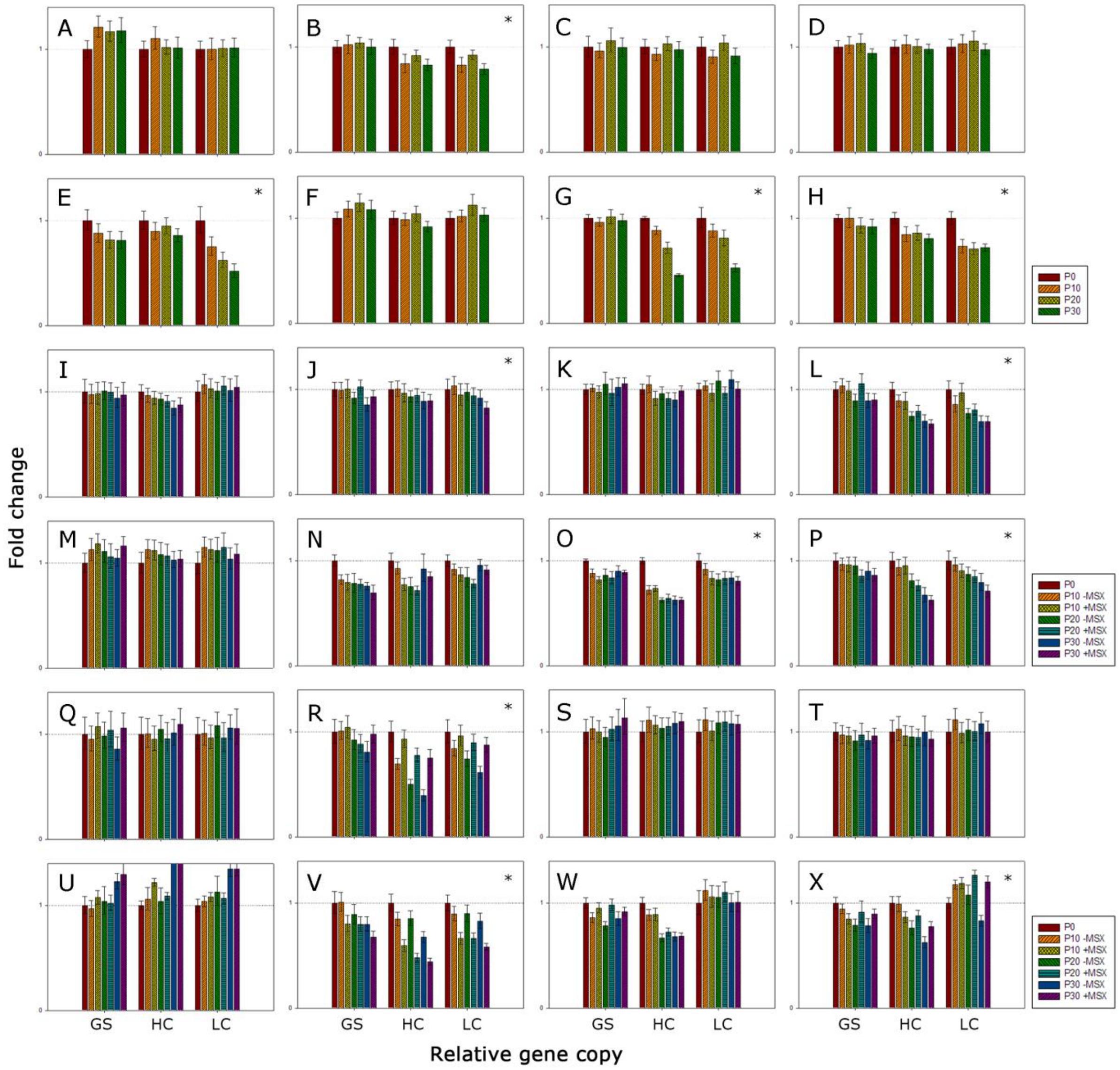

Supplementary Table S1. Analysis of the 24 high producing clones selected at various MSX concentrations in the cultures with or without MSX. Values for the gene copy number and mRNA level are relative to those of K1-0 2-3.

Clones	Host cell line	MSX (μM)	μ (/day)	qP (pg/cell/day)	Gene copy number (relative to K1-0 2-3)			mRNA level (relative to K1-0 2-3)		
					GS	HC	LC	GS	HC	LC
K1-0 1-1	CHO-K1	0	0.70 ± 0.02	13.9 ± 0.8	1.4	5.3	2.3	15.6	3.1	5.2
K1-0 1-2	CHO-K1	0	0.72 ± 0.06	10.3 ± 1.0	1.5	5.4	2.6	13.0	2.3	3.7
K1-0 2-1	CHO-K1	0	0.68 ± 0.05	6.0 ± 0.6	1.0	1.9	2.1	0.9	1.2	1.2
K1-0 2-3	CHO-K1	0	0.70 ± 0.03	3.0 ± 0.7	1.0	1.0	1.0	1.0	1.0	1.0
KO-0 1-1	GS KO	0	0.76 ± 0.06	11.7 ± 1.7	1.6	2.1	4.1	35.5	4.4	13.7
KO-0 1-3	GS KO	0	0.78 ± 0.02	7.0 ± 0.6	1.2	2.0	2.2	16.3	2.6	3.9
KO-0 2-1	GS KO	0	0.59 ± 0.03	37.3 ± 2.6	0.7	1.7	2.6	17.1	11.2	22.6
KO-0 2-2	GS KO	0	0.55 ± 0.05	39.2 ± 2.8	0.7	1.2	2.7	20.6	10.5	53.3
K1-25 1-1	CHO-K1	25	0.75 ± 0.01	19.5 ± 0.8	3.3	15.0	17.3	38.5	7.2	13.2
K1-25 1-2	CHO-K1	25	0.73 ± 0.02	15.9 ± 0.4	3.0	13.5	19.5	48.7	6.9	11.0
K1-25 2-1	CHO-K1	25	0.60 ± 0.01	22.9 ± 2.1	1.2	2.1	2.0	19.7	2.8	5.8
K1-25 2-2	CHO-K1	25	0.77 ± 0.06	10.8 ± 1.3	1.5	3.0	3.1	20.7	3.7	4.9
KO-25 1-1	GS KO	25	0.57 ± 0.05	35.1 ± 2.7	3.9	11.4	15.8	43.7	5.7	19.3
KO-25 1-3	GS KO	25	0.68 ± 0.01	18.6 ± 1.8	1.1	1.0	4.0	17.3	3.9	12.1
KO-25 2-1	GS KO	25	0.60 ± 0.04	28.5 ± 0.5	0.7	1.7	4.5	18.8	14.9	27.2
KO-25 2-2	GS KO	25	0.65 ± 0.05	30.5 ± 0.5	0.8	3.3	4.7	17.0	10.5	20.1
K1-50 1-2	CHO-K1	50	0.53 ± 0.02	31.7 ± 0.8	1.5	1.7	3.0	12.3	3.5	8.1
K1-50 1-3	CHO-K1	50	0.59 ± 0.07	24.8 ± 4.9	1.5	1.5	3.0	36.2	2.7	11.2
K1-50 2-1	CHO-K1	50	0.61 ± 0.02	23.5 ± 2.0	6.3	32.9	35.0	21.8	7.6	13.3
K1-50 2-2	CHO-K1	50	0.59 ± 0.01	14.3 ± 0.5	6.2	32.2	32.5	38.6	6.3	9.7
KO-50 1-1	GS KO	50	0.53 ± 0.04	11.6 ± 1.5	1.6	4.5	3.5	48.1	1.3	2.2
KO-50 1-3	GS KO	50	0.72 ± 0.08	4.7 ± 0.7	1.3	3.3	3.1	31.6	0.7	2.4
KO-50 2-1	GS KO	50	0.48 ± 0.03	15.6 ± 1.2	0.7	2.3	2.0	8.7	1.6	1.1
KO-50 2-2	GS KO	50	0.49 ± 0.01	14.3 ± 1.0	0.6	2.2	2.1	7.2	3.6	3.0

Supplementary Table S2. Analysis of the clones selected at various MSX concentrations during long-term cultures with or without MSX..

Clones	Host cell line	MSX level (μM)	Presence of MSX	Percent change in μ (%)	Percent change in q_p (%)	Percent change in relative gene copy number (%)		
						GS	HC	LC
K1-0 1-1	CHO-K1	0	-	5.7	-40.3	22.0	1.2	1.3
K1-0 1-2 *	CHO-K1	0	-	3.3	-34.8	-0.1	-17.1	-21.2
K1-0 2-1	CHO-K1	0	-	7.8	-97.4	0.3	-2.7	-8.7
K1-0 2-3	CHO-K1	0	-	0.4	-42.8	-6.2	-2.1	-2.4
KO-0 1-1 *	GS KO	0	-	6.1	-72.9	-18.9	-14.2	-48.1
KO-0 1-3	GS KO	0	-	6.0	-10.6	8.0	-8.0	3.1
KO-0 2-1 *	GS KO	0	-	12.5	-43.5	-2.0	-54.2	-47.5
KO-0 2-2 *	GS KO	0	-	26.7	-43.3	-8.1	-19.5	-28.2
K1-25 1-1	CHO-K1	25	-	7.5	-45.7	-5.9	-15.6	1.2
			+	8.5	-35.9	-2.9	-12.7	4.2
K1-25 1-2 *	CHO-K1	25	-	9.2	-32.1	-14.6	-11.0	-8.1
			+	12.7	-13.6	-7.0	-10.8	-17.6
K1-25 2-1	CHO-K1	25	-	29.8	-58.5	2.3	-10.0	9.4
			+	24.3	-53.5	5.5	-1.3	0.3
K1-25 2-2 *	CHO-K1	25	-	6.1	-40.0	-10.7	-30.3	-30.4
			+	2.6	-34.9	-9.8	-32.6	-30.8
KO-25 1-1	GS KO	25	-	33.2	-58.9	4.8	3.0	3.8
			+	32.9	-42.3	19.8	7.1	12.1
KO-25 1-3	GS KO	25	-	16.3	-46.2	0.7	0.3	7.9
			+	15.0	-29.1	-2.0	17.2	8.9
KO-25 2-1 *	GS KO	25	-	15.3	-42.2	-10.1	-37.5	-16.4
			+	14.8	-36.5	-11.1	-37.5	-19.4
KO-25 2-2 *	GS KO	25	-	7.1	-44.1	-10.0	-32.6	-20.5
			+	7.6	-46.1	-13.7	-37.6	-29.1
K1-50 1-2	CHO-K1	50	-	40.6	-31.8	6.2	14.8	24.8
			+	26.8	-28.0	10.7	8.8	18.9
K1-50 1-3 *	CHO-K1	50	-	25.9	-80.4	-19.2	-60.9	-38.7
			+	19.9	-58.5	-2.3	-25.1	-12.7
K1-50 2-1	CHO-K1	50	-	15.3	-47.5	5.6	8.1	7.8
			+	18.4	-17.5	13.4	9.7	7.1
K1-50 2-2	CHO-K1	50	-	19.4	-62.1	-8.3	-0.2	10.3
			+	22.9	-32.0	-3.9	-6.9	-0.1
KO-50 1-1	GS KO	50	-	36.5	-26.1	25.5	67.6	37.7
			+	35.1	-32.3	27.2	46.6	32.5
KO-50 1-3 *	GS KO	50	-	5.4	-49.9	-20.1	-32.4	-17.3
			+	3.7	-50.6	-32.5	-56.0	-41.6
KO-50 2-1	GS KO	50	-	27.1	-5.2	-8.1	-31.9	8.3
			+	22.1	8.3	-8.0	-31.5	9.1
KO-50 2-2 *	GS KO	50	-	14.6	-25.4	-20.2	-35.1	-17.1
			+	17.9	50.0	-8.5	-19.5	22.7

The % change in μ and q_{mAb} was calculated based on the average values of the first and last three batches during the long-term cultures. The % change in the relative gene copy number of GS, HC and LC was calculated based on the values of the first and last batches during the long-term cultures. Asterisk (*) indicates clones which showed more than a 10% change in relative gene copy number of more than two genes among GS, HC, and LC during the long-term cultures

Supplementary Table S3. Primer sequences used in PCR reactions.

Name	Sequences (5' → 3')
GAPDH forward	GGA CAT CAA GAA GGT GGT GAA
GAPDH reverse	GAG TGG GAG TCA CTG TTG AAG
GS forward	CCA AAT AGG ACC CTG TGA AG
GS reverse	GGT GCT AAA GTT GGT ATG GC
HC forward	CAG CCG GAG AAC AAC TAC AA
HC reverse	CAT CAC GGA GCA TGA GAA GA
LC forward	GTT GTG TGC CTG CTG AAT AAC
LC reverse	TCC TGC TCT GTG ACA CTC T

The qPCR reactions were incubated at 95 ℃ for 5 min and then cycled 39 times at 95℃ for 10 sec, 58℃ for 30 sec and 72℃ for 30 sec.
